# Virtual Recovery of Content from X-Ray Micro-Tomography Scans of Damaged Historic Scrolls

**DOI:** 10.1038/s41598-018-29037-x

**Published:** 2018-08-09

**Authors:** Paul L. Rosin, Yu-Kun Lai, Chang Liu, Graham R. Davis, David Mills, Gary Tuson, Yuki Russell

**Affiliations:** 1 School of Computer Science & Informatics, Cardiff University, Queens Buildings, 5 The Parade, Cardiff, CF24 3AA UK; 2 School of Astronautics, Beihang University, Beijing, 100191 China; 3 Institute of Dentistry, Queen Mary University of London, Francis Bancroft Building, Mile End Road, London, E1 4NS UK; 4 Norfolk Record Office, The Archive Centre, Martineau Lane, Norwich, NR1 2DQ UK

## Abstract

There is a large body of historical documents that are too fragile to be opened or unrolled, making their contents inaccessible. Recent improvements in X-ray scanning technology and computer vision techniques make it possible to perform a “virtual” unrolling of such documents. We describe a novel technique to process a stack of 3D X-ray images to identify the surface of parchment scrolls, unroll them, and create a visualization of their written contents. Unlike existing techniques, we can handle even challenging cases with minimal manual interaction. Our novel approach was deployed on two 15th and 16th century damaged historic scrolls from the manors of Bressingham and Diss Heywood. The former has become fused, probably due to exposure to moisture, and cannot be fully unrolled. The latter was severely burnt several hundred years ago, becoming thoroughly charred, heat-shrunken, and distorted, with all the sheets now brittle and fused together. Our virtual unrolling revealed text that has been hidden for centuries.

## Introduction

That archivists ‘should protect the integrity and reliability of archival material and thus guarantee that it continues to provide reliable evidence of the past’ is fundamental to their code of ethics^1^. This presents a particular problem when unique (as almost all archives are) information is locked away in badly damaged documents. Should an attempt be made to open a document when there is a risk that some of the information it contains will be lost? Alternatively, should the document be left untouched in the hope that a new way of getting at the information it contains, no matter how unlikely, will be found? Now, those who ‘protected the integrity’ of those documents by putting their trust in the future look to have been proven right as new techniques show that there may be a way in which these records can reveal their secrets.

Before documents arrive in a Record Office, where they can be stored in a temperature and humidity controlled environment, they are often stored in far from suitable conditions. Basements, lofts, sheds and garages are often damp and sometime just plain wet. In these conditions, documents age quickly as they are simultaneously subjected to mould attack, mechanical damage and chemical deterioration. In the worst cases short of permanent loss, paper turns to a friable mass held together by gravity and ink; parchment distorts, discolours, becomes brittle and gelatinizes, turning into a solid mass of glue, animal skin and ink. At the other end of the damage spectrum is fire. Obviously, this usually leads to total loss, but sometimes documents survive as a discoloured, distorted, fused mass. This is not an unusual problem; many Record Offices in the UK hold parish registers scorched during the blitz, their welded pages hiding the only available information on thousands of people who lived in the sixteenth, seventeenth and early eighteenth centuries.

In recent years there has been a burst of research activity with the goal of using X-ray microtomography (XMT or micro-CT) to perform volumetric scanning of delicate and damaged historical documents such as parchments or papyri in order to provide a non-invasive solution to extracting and visualising their contents. XMT is a miniaturised version of medical human body CT scanning, designed to look at specimens much smaller than the human body, with a resolution in the region of microns, and the X-ray absorption contrast is strong for iron gall ink, which is the most commonly used ink in Europe between the 12th and 19th centuries. Moreover, spectroscopy and X-ray diffraction were used to investigate the effects of XMT scanning on the parchment structure, and experiments did not find evidence of any damage or change in the material properties^2^, indicating the suitability of XMT for parchment analysis.

A pioneer on scanning parchments has been Brent Seales, although his results have been limited until recently. His early work only scanned replicas and experimental artifacts^3^. In Baumann *et al*.^4^ he scanned a small portion of a 15th century manuscript of Ecclesiastes, which consisted of seven or so layers that were stuck together. However, results were only shown for the top layer, and there was no mention of the (manual or automatic) segmentation stage. Seales *et al*.^5^ describe how two Herculaneum papyri were scanned. However, their subsequent processing required time consuming manual segmentation, which meant that only small portions of the scroll were viewed, and they contained no visible text or ink. Recent work has succeeded in revealing the contents of a charred scroll from En-Gedi^6^. A semi-automatic segmentation process was developed that enabled him to process much larger amounts of data than before, but it still requires substantial manual interaction. Given the age of this scroll, the ink contrast appears to be uncharacteristically high, making textual extraction a little easier, though this is still an impressive result.

Whilst there has been much attention to improving spatial resolution and scanning speed of XMT scanners, little has been done to improve contrast resolution and elimination of artefacts. Successful text recovery depends firstly on obtaining a good clean scan, with good signal-to-noise ratio and free from artefacts as far as possible. The MuCAT scanner at Queen Mary University of London is able to deliver very high-quality scans, though at the cost of scanning speed. However, compared with the logistical arrangements for scanning an archived scroll, neither scanning nor computation are bottlenecks.

In the last few years many other research groups have been working on the topic of recovery of information from scanned documents, many using synchrotron radiation micro-CT. However, most are limited in various ways, such as: proof of concept systems still operating with phantoms or simple test material^7–11^; scanning without attempting to segment the artifact^12–14^; processing artifacts with sufficiently simple geometry that the segmentation can be performed with minimal manual interaction^15^; restricting attention to small portions of the data so that manual interaction becomes feasible^16,17^.

Thus, while many of the research groups have focussed on developing the scanning technology, which has become relatively mature, the subsequent image processing steps have become the bottleneck in being able to scale up to coping with large data sets and complicated geometry. The standard processing pipeline involves the following basic components: scanning, parchment/air separation, parchment layer segmentation, surface flattening, ink projection. It is the parchment layer segmentation that causes the greatest difficulty due to the fusing of layers due to physical damage, compression, scanning limitations, etc. Since there may be no actual separation between the layers in the data, their segmentation is challenging, and needs to consider the local and global geometry.

Samko *et al*.^18^ took a step towards addressing this technology gap, developing a novel graph cut method with a shape prior in order to extract and separate parchment layers. They demonstrated their method on several historic parchment scrolls, but they had relatively simple geometry. Subsequently this work was improved by Liu *et al*.^19^ who were the first to tackle the 15th century Bressingham scroll which was significantly more challenging due to the complicated geometry. Not only were layers stuck together such that their separating boundary information had to be reconstructed, but also some layers were broken, and these gaps also had to be reconstructed to resynthesise the connections between sections of parchment. While Liu *et al*. were successful in performing unrolling to reveal the text from 3070 slices, this only constitutes about a third of the scroll. Their technique was unable to cope with the remainder of the Bressingham scroll or the Diss Heywood burnt scroll, since this technique has several drawbacks. First, it is based on the assumption that the thickness of the parchment layer is identical everywhere and known a priori. This prerequisite is not met by parchments which have suffered mistreatment such as compression and distortion. Second, this method separates a region made of several fused layers by a curve which is as parallel as possible to the preserved boundary without consideration for the region shape. Consequently, if the fused region is too thin, the as-parallel-as-possible curve will touch the layer boundary and thus break the entire layer into several fragments. Therefore a re-linking process is indispensable for this method. However, it is easy for the re-linking to make topological errors when there are so many fragments. Finally, this technique does not make use of the information of the previous processed slice to initialise the current segmentation, which can quite easily lead to segmentation errors if many layers are merged in a long and thin fused region. The work described in this paper is an extension of Liu *et al*.’s method, but the pipeline of the segmentation algorithm in this work is entirely novel. In this paper, both the segmentation initialisation and the broken layer connection are conducted based upon the processing result of the previous slice, which makes the proposed method much more accurate and robust than Liu *et al*.’s method. In addition, our segmentation algorithm separates the fused region into several layers as evenly as possible without the assumptions of identical layer thickness and prior knowledge of the thickness of the parchment layers which were required by Liu *et al*.’s method, so that our method will not fragment the entire layers. The segmentation result is finally refined with the skeleton of the layer in the previous processed slice, which effectively ensures the consistency of the topological structure of the scroll layer. Accordingly, the proposed approach advances Liu *et al*.’s method such that both of these challenging scrolls were completely unrolled. The experimental comparison of the proposed and Liu *et al*.’s methods is provided in SI. New contributions of this paper include: improved skeleton propagation between images, an optimization method for even segmentation of an arbitrary number of fused layers, and the processing of multiple sheets.

## Results

Our novel approach was deployed on two historic 15th and 16th century scrolls from the manors of Bressingham and Diss Heywood. Texts on parchments from this era were typically written with iron gall ink, and its high density provides a strong X-ray contrast between ink and the parchment, making successful text recovery feasible. A bathophenanthroline paper test confirmed that both the Bressingham and Diss Heywood scrolls were written with iron gall ink.

### Bressingham Scroll

**Figure 1:**
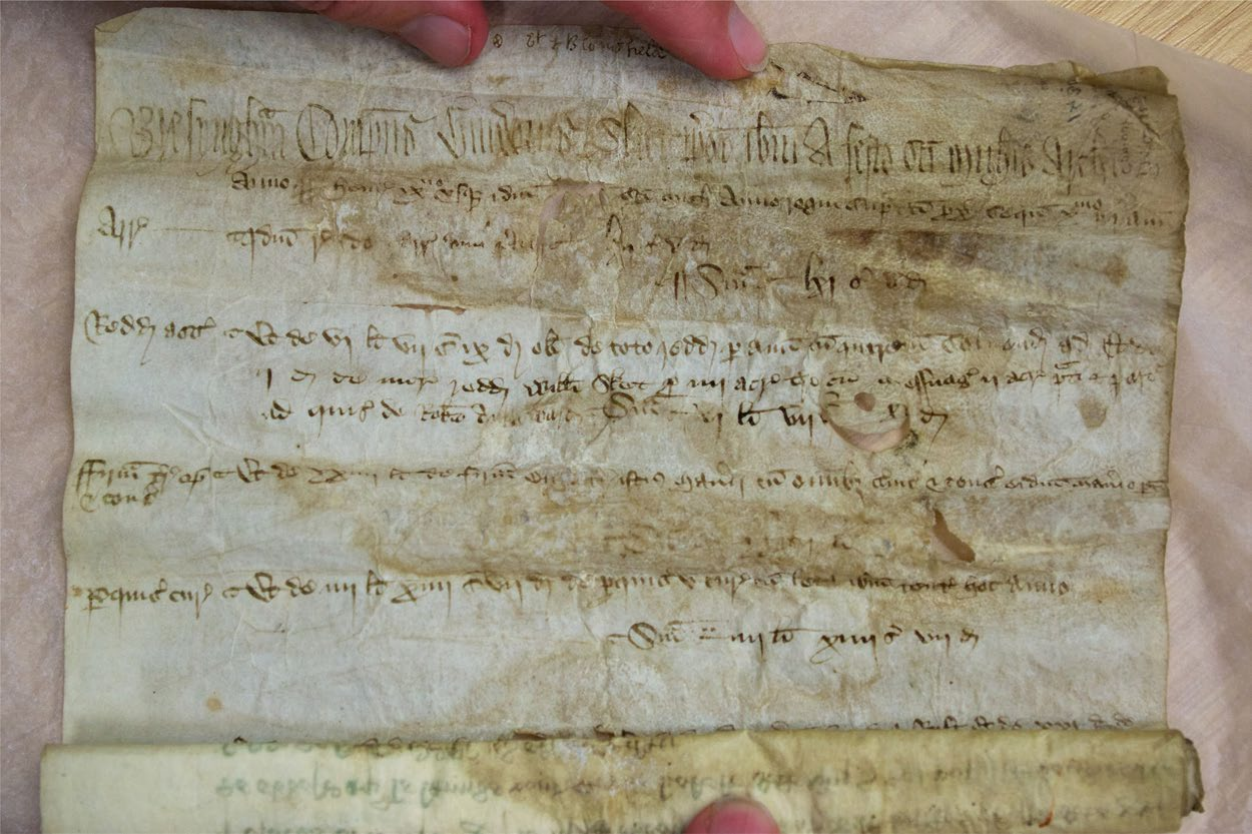
The 15th century Bressingham scroll, which can only be partially unrolled.

The Bressingham scroll (see Fig. 1) is an account from the manor of Bressingham, dated 1408-9 (NRO, PHI 468/5). The records on the scroll include: the income of the lord from the manor and his expenditure, profits from holding the manor court, sales of underwood, and leasing out the fishing rights. The width of the scroll is approximately 270 mm though the total length is unknown, as it is impossible to unroll completely in a safe manner: approximately only the first 100 mm of the parchment is accessible as the rest of the scroll has become fused together. The accessible part of the text revealed that the manuscript is written in iron gall ink. The fusing of the scroll is most likely to have been caused by exposure to moisture while being stored in damp storage before the scroll was deposited with Norfolk Record Office (NRO). It may be possible to unroll the scroll slightly further with some interventive treatment. However, the fusion indicates that it would be impossible to unroll the scroll completely to the end without damaging the parchment, and would most likely cause delamination, tear, loss of the material and possibly permanent change of the surface appearance (depending on the method used for unrolling). Indeed, there are several areas of the parchment, which have aleady become abraded, delaminated and torn. This type of damage suggests previous attempts to unroll the scroll after it became fused together. Despite the desire to access further text hidden in the fused portion, there is currently no conservation method which could enable safe unrolling of the entire scroll. Therefore the scroll has remained unconserved and hence, inaccessible. Given this situation, NRO had little choice but to investigate the possibility of a non-interventive option in order to access the remaining text.

**Figure 2:**
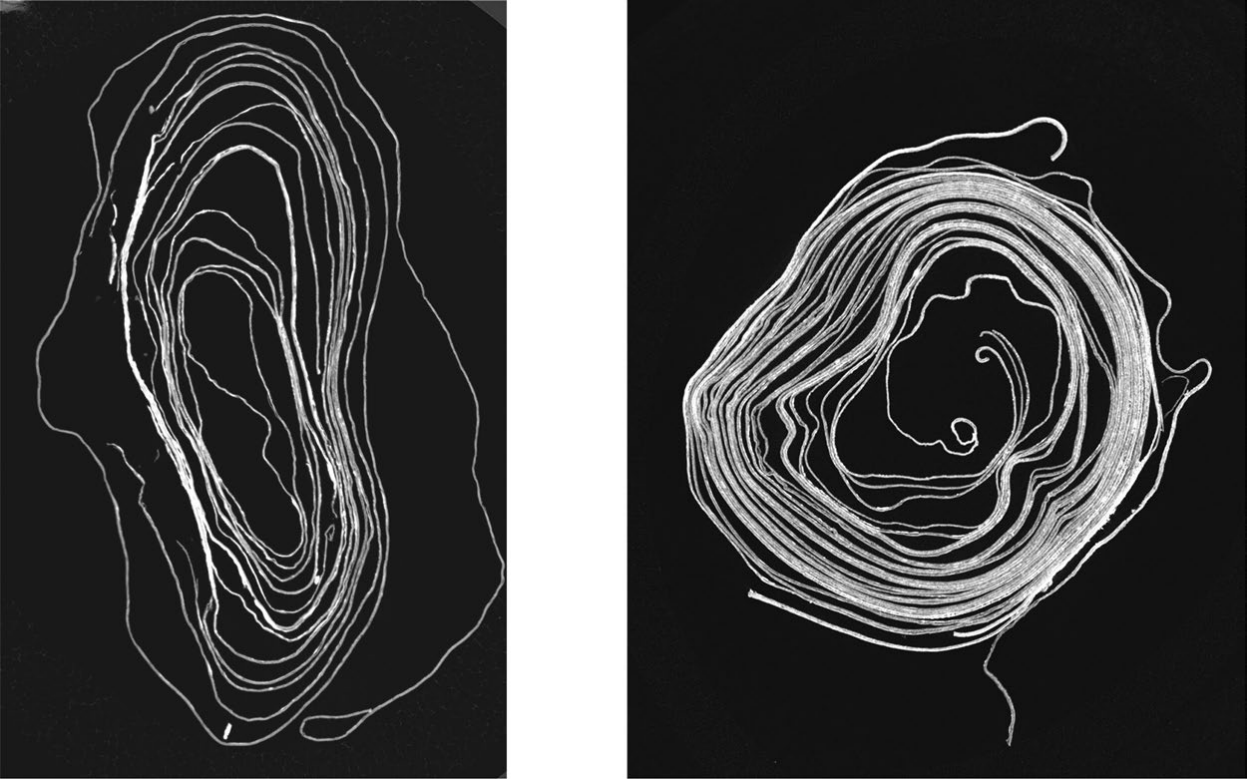
Slices from XMT scans of scrolls: (**a**) Bressingham, (**b**) Diss Heywood.

**Figure 3:**
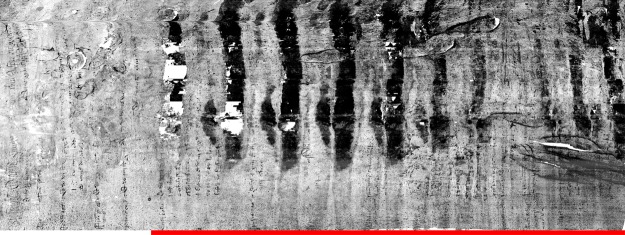
The results of applying virtual unrolling to the Bressingham scroll. The red line indicates the section of the scroll that cannot be physically unrolled without incurring damage.

Figure 2a shows an example slice from the XMT scan in which the fusing of several layers is evident. The results of applying our virtual unrolling pipeline to the Bressingham scroll are shown in Fig. 3, and has enabled the following interpretation. This Manorial Account from the Bressingham in Norfolk is, as its heading identifies, a bailiff’s account dated Michaelmas 9 Henry IV: this means it is from 1409. It is an example of what PDA Harvey (Manorial Records) calls a ‘Phase 3’ Account where the manor has been farmed out for a set amount – £24 a year we learn from the scan. This type of account does not detail the crops and livestock but it does show that the lord of the manor retained some woodland and fisheries from which income accrued. The first half of the account is of income, the total is marked Summa half way down. The second, less legible half, is of expenses and includes expenses for the house, stewards expenses (for holding courts etc.), fees and allowances. This would have resulted in a total for the year which would show the profit, or loss, but this cannot be seen.

**Figure 4:**
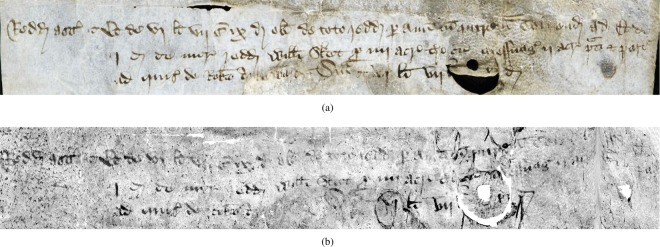
A section of the Bressingham scroll that is (**a**) visible after partial unrolling, and (**b**) the result recovered from the XMT scan.

To validate the correctness of the virtual unrolling, Fig. 4 provides a comparison of a portion of the visible section of the scroll along with the recovered contents. It can be seen that most of the text has been successfully recovered from the XMT scan, although there is some loss of ink contrast as well as geometric distortion (i.e., the lines of text are bent in the middle). The reduced ink contrast is evident in the raw XMT scan, indicating that it is not an artifact of the segmentation or ink projection stages, but instead is caused by reduced ink density, e.g. due to the pen running low on ink. The geometric distortion is caused by inaccurate skeleton positions, especially due to holes or fused layers in the parchment where skeleton locations are under-constrained from the image information.

The fused section of the scroll are compacted, and appear brighter than the unfused sections in the XMT scans, see Fig. 2a. This subsequently leads to these dense sections appearing as the large dark regions in Fig. 3, in which all ink content has been lost. In addition, there are holes in the parchment, which appear as the empty white regions in Fig. 3. Unfortunately these factors significantly reduced the amount of recovered text: overall, about 15–30% of readable text is obtained.

### Diss Heywood Burnt Scroll

**Figure 5:**
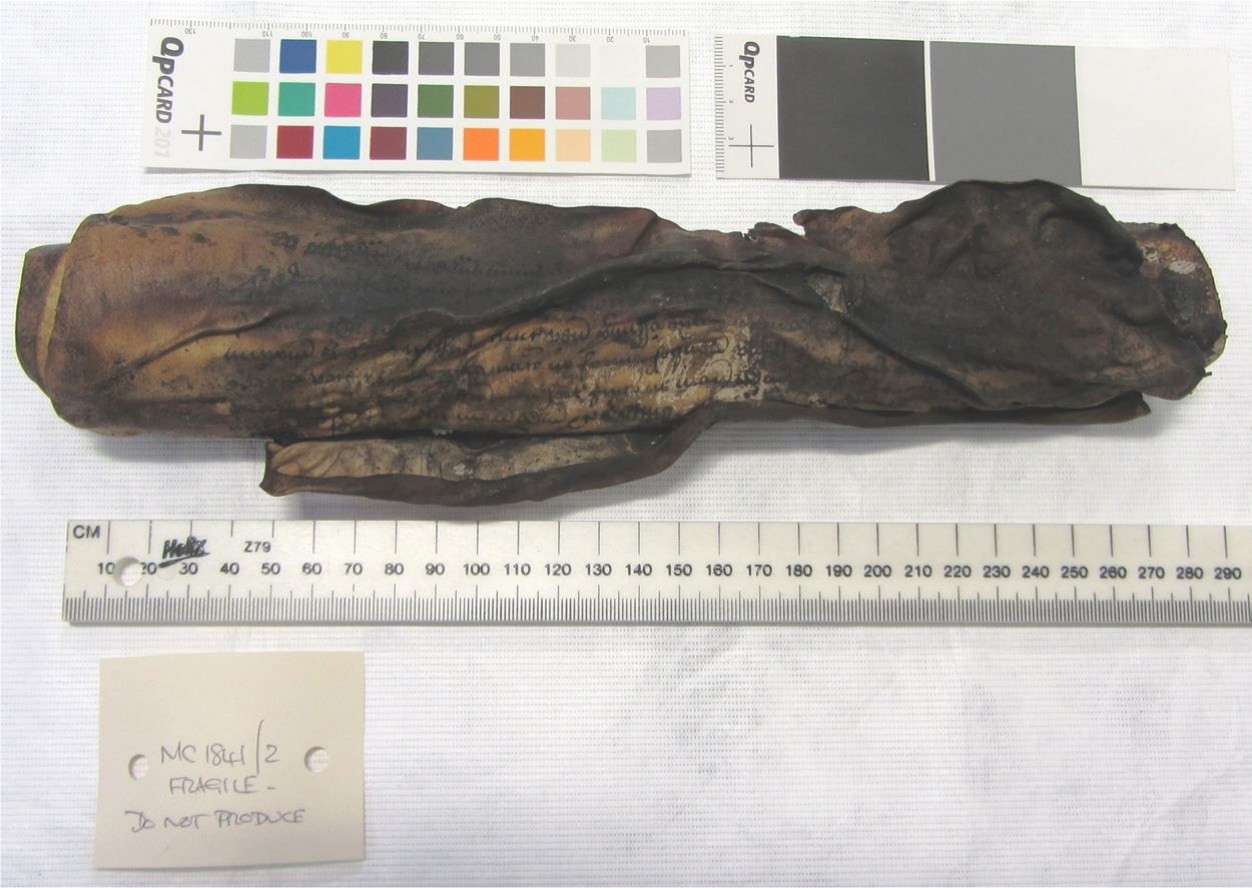
The 16th century Diss Heywood scroll, which is severely charred.

The 16th century Diss Heywood burnt scroll (Norfolk Record Office MC1841/2) is shown in Fig. 5, and is a court roll from Diss Heywood Manor in Norfolk that has been damaged by fire. This roll, dating from the 16th century, will contain information on life in the manor as it was administered by its manorial court. There may be details of manorial services, land transactions, disturbances of the peace, payment of fines, names of jurors and information on the upkeep of land. This type of information that can be used to study demography, social history, agricultural yields and much more. However, the poor condition the document is in means that it is impossible to tell exactly what it contains.

The parchment scroll is approximately 270 mm wide; the total length is unknown as it is impossible to unroll. The item is in extremely poor condition resembling a giant cigar due to obvious fire and heat exposure. The whole scroll has been solidly fused together - the extremely firm texture and blackened sides of the scroll suggest significant damage extends through all layers. Although the whole scroll is firm, brittleness of the parchment has made safe handling extremely difficult, and so a virtual unrolling is the only practical option. While it can be seen that the manuscript has text on both sides of the membranes in dark ink (likely to be iron gall ink), it is impossible to extract the written information due to a heavily discoloured and creased surface and soot like deposits adhered over the entire outer layer of the scroll.

Generally, heat-deteriorated parchments are drastically shrunken. However, judging by the size of the letters visible on the surface, this does not appear to be the case with this particular parchment scroll.

**Figure 6:**
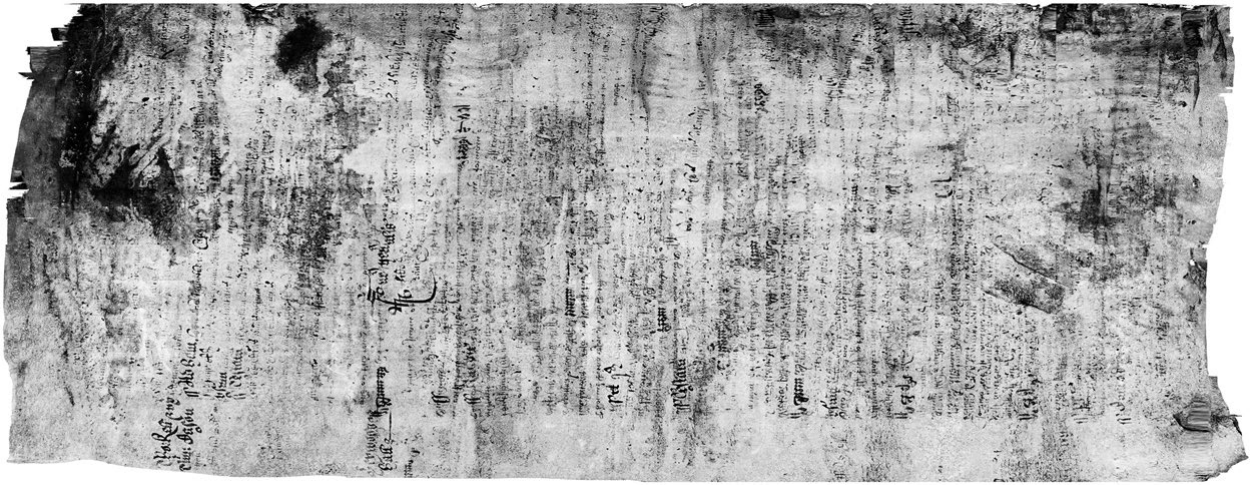
The results of applying virtual unrolling to one of the sheets of the Diss Heywood burnt scroll.

The XMT scan shown in Fig. 2b reveals that the Diss Heywood scroll consists of four sheets which are tightly wound together with many touching layers, posing a challenge to the segmentation process. In contrast to the Bressingham scroll, which only contains writing on one side of a single sheet of parchment, it becomes problematic for the Diss Heywood scroll to separate text written on adjacent sheets. When these sheets are touching, then even minor inaccuracies in segmentation will cause text to be assigned to the wrong sheet. This is evident in the results of applying our virtual unrolling pipeline to the Diss Heywood scroll shown in Fig. 6. The result visualises one sheet, but in fact it also contains text derived from the adjacent sheet which has been superimposed.

**Figure 7:**
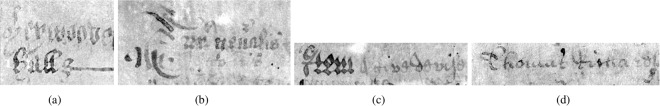
Excerpts from the virtual unrolling of the Diss Heywood scroll.

Nevertheless, it is possible to start to pick out some pieces of information. Importantly, it confirms that it does indeed relate to Heywood Hall (see Fig. 7a) and that they are records of the Curia Generalis (Fig. 7b), the General Court, which usually refers to the Court Leet where peace keeping functions were exercised. The scan also confirms that the scroll also deals with land transactions and possibly testamentary business. On one of the few part written in English it clearly states, “Item I give devise”, see Fig. 7c. It is also possible to start picking out names, for example Fig. 7d contains Thomas Richards, or possibly Richardson.

## Discussion

Theoretically and experimentally, our method can provide correct segmentation for a scroll as long as the layers are more than 1 voxel thick. The thickness of the Diss Heywood scroll layers is around 7 voxels, with the thinnest portion being only about 3 voxels thick. Although the Bressingham scroll layers are approximately 5 voxels thick on average, in places where the parchment is seriously abraded and delaminated the layer only has about 0.5 voxels. Therefore, in order to apply our algorithm we first resized the Bressingham scroll to double the original width and height before processing. Even after resizing, the thinnest portions of the Bressingham scroll layer are still only 1 voxel thick. Despite this, our method was still able to generate the correct segmentation for these two scrolls, as demonstrated in the experiment in the SI.

Each scroll was scanned as several (2 or 4) volumes, and for each volume our segmentation method started with a slice that was manually segmented as initialisation. New initialisations were need for each volume since the overlap and physical movement of the scroll during scanning made it difficult to propagate the segmentation result from one volume to its adjacent one. Apart from these initialisations, the segmentation process was essentially automatic, only needing manual correction on 14 (out of 9938) slices for the Bressingham scroll due to serious damage in fused regions, and 15 (out of 8044) slices for the Diss Heywood scroll where a large part of the parchment has disappeared.

Run-time for our segmentation method is computationally expensive: requiring approximately 4 minutes per slice, so that segmenting each scroll took about 3 weeks. However, compared to other methods which need frequent manual interactions during processing, our method can still be thought of as relatively efficient. Moreover, currently the code is unoptimised Matlab, the efficiency of which could be substantially improved.

The results shown in this paper demonstrate that the proposed method is capable of performing a virtual unrolling of extremely challenging data sets in a mostly automatic manner. This paves the way to a larger scale application of the software pipeline to a larger range of documents, including those of greater historic significance.

One application of the technology is to use it to determine if conservation work on a particular item might be worthwhile. Sometimes archives have records which require extensive and costly conservation work but, beyond the fact that they are known to be old, the archivists have no idea of what information they contain. Thus, our virtual unrolling approach can provide an indication of a document’s contents, enabling archivists to make better decisions on work prioritization and better support funding applications for conservation work.

## Methods

### XMT

The scrolls were scanned using the high contrast MuCAT 2 XMT scanner designed and run at Queen Mary University of London^20^. This system employs a cooled CCD camera in time-delay integration mode (reading out whilst moving the camera) in order to maximise the potential signal-to-noise ratio and minimise artefacts. The system uses a 225 kV microfocus X-ray generator from X-tek (now part of Nikon Metrology, Tring, Hertfordshire, UK) and an 800 series cooled CCD camera from Spectral Instruments (Tucson, Arizona), fiber-optically coupled to a columnated CsI scintillator from Applied Scintillation Technologies (now Scintacor, Cambridge, UK). The source to scintillator distance was 24 cm. The 16 megapixel Fairchild CCD485 sensor was binned 2 × 2 in hardware and a further 2 × 2 after TDI readout to give an effective 60 μm pixel size (appropriate for the scintillator resolution). The ultimate reconstructed voxel size can be adjusted from 5 to 40 μm by setting the source to sample distance appropriately. Although the camera was capable of recording 1024 image rows, only an 800 row region was used due to unacceptable distortion in the fiber-optic faceplate in the lowest portion. The use of TDI imaging meant that the image width could be adjusted up to the maximum field of the X-ray beam.

For the Bressingham scroll, it was set to 30 kV and 540 μA and the geometric magnification was set to give a 30 μm voxel size. The scroll was wrapped and tied in a Plastazote sheath prior to transportation from the Norfolk Records Office. For scanning, it was placed in a polypropylene container to keep it in place. The scanner was initially set to record 2007 X-ray projections around 360°, after which the scroll was automatically lowered and another set of projections recorded. This was repeated to record 12 “blocks” of projections. Because of limited vertical travel, the scroll was then turned upside down and a further 5 blocks scanned, such that a small overlap should occur in the scanned regions. Each block took around 7 hours to record. After scanning, the projections were corrected for beam-hardening and reconstructed using a standard cone-beam back-projection algorithm. Where successive blocks were recorded consecutively, the resultant reconstructions were concatenated to form a single volume. However, due to unexpected interruptions in the automated scanning process, four separate volumes were created, meaning that the final joining would be performed after virtual unrolling. The 32-bit volumes were “trimmed” to the smallest cuboid that contained the scroll and reduced to a one-byte-per-voxel data format. The four volumes had dimensions of 1186 × 966 × 1228, 1226 × 826 × 2456, 1356 × 770 × 3684, and 1256 × 816 × 3070 voxels.

The Diss Heywood burnt scroll was scanned at 40 kV and 405 μA with a 35 μm voxel size. The scroll was first placed in an open cylindrical plastic container of about half its height (with the long axis vertical). The scroll was packed lightly with cut sheets of Plastazote foam to keep it from moving during the scan. The scanner was set to record 2511 X-ray projections per block for 7 blocks, taking around 3 days. The scroll was then turned upside down and a further 7 blocks scanned, such that a small overlap should occur in the scanned regions. After reconstruction and trimming, the final 2 volumes had dimensions of 1780 × 1830 × 4012 and 1480 × 1880 × 4032 voxels.

### Virtual Unrolling

We assume that the scrolls do not contain significant large scale deformation, i.e., the scroll can be scanned such that 2D images sampled along one of the axes of the 3D volume contain cross-sections along the length of the scroll (as shown in the examples in Fig. 2). This assumption is reasonable for typical scrolls such as those analysed in this paper. The presence of large scale deformation would require estimating the local direction of the scroll in order to orient the cross-section images appropriately.

There is usually a significant difference between the densities of parchment and air in the X-ray tomographic images. Therefore, the processing to extract their content starts with performing an initial segmentation using thresholding^21^. While the overall segmentation is reasonable, it will contain many local errors due to fused sections of parchment or holes in the parchment. These are subsequently corrected by the following steps, in which the images comprising the stack are processed in sequence.

Junctions – which indicate fused layers – are detected in each image from the segmentation boundaries.

The skeletons of the foreground and background of the previous image are propagated to the current (adjacent) image and used to match the pairs of junction sections which should be connected, and provide an initial guess to the reconstruction of the missing boundary curves.

We assume that the thickness of the parchment layers should be approximately locally uniform. And so for regions of the parchment that contain multiple fused layers, the locations of reconstructed missing boundary curves are optimized in order to separate the fused region into several layers as evenly as possible.

For images in which layers of the parchment are broken or missing, the skeleton segments from the previously processed image are used to determined how to connect any gaps.

Finally, the text of the parchment is revealed by surface modelling and ink projection.

We give details of these steps in the following sections.

### Propagation

**Figure 8:**
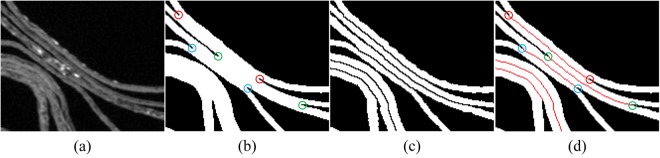
The fused region in a parchment slice. (**a**) A fused region of the slice *i*; (**b**) the initial segmentation ${{\bf{\text{S}}}}^{0}{}_{i}$ of the slice *i*, on which the matched junction sections are circled in the same color; (**c**) the final segmentation **S**_*i*−1_ of the slice *i*−1, and (**d**) the Boolean intersection **C**_*i*_ of the skeleton of the background of **S**_*i*−1_ and ${{\bf{\text{S}}}}^{0}{}_{i}$.

^1^

Figure 8a,b demonstrate a typical fused region in the slice *i* as well as its initial segmentation ${{\bf{\text{S}}}}^{0}{}_{i}$ generated by the Otsu method^21^. As can be seen, between the two fused layers there must exist a pair of corners on the layer boundaries, which are circled in the same color in Fig. 8b. Such corners are called junction sections, and are detected by the approach described in^19^. In the sequence of the parchment slices, any two adjacent slices are very similar, so we will propagate the previous segmentation result to the next slice to implement the junction section matching and initialise the optimization in the next section. Given the final segmentation **S**_*i*−1_ of the slice *i*−1 (Fig. 8c), the skeleton of the background of **S**_*i*−1_ which is included in the foreground of ${{\bf{\text{S}}}}^{0}{}_{i}$ can be calculated by Boolean intersection

1 $${{\bf{\text{C}}}}_{i}={\text{skeleton}}({\bar{{\bf{S}}}}_{i-1})\cap {{\bf{S}}}^{0}{}_{i},$$

where skeleton (.) represents the morphological skeleton extraction, and ${\bar{{\bf{S}}}}_{i-1}$ denotes the background of **S**_*i*−1_. The obtained curves in **C**_*i*_ are superimposed upon ${{\bf{S}}}^{0}{}_{i}$ and illustrated in Fig. 8d. As can be seen, a pair of junction sections which should be matched are close to a curve in **C**_*i*_. Therefore, the curves in **C**_*i*_ can be used to match the pairs of junction sections which should be connected, and will provide an initial guess for the final segmentation.

However, the narrow fused region in ${{\bf{\text{S}}}}^{0}{}_{i}$ may obstruct the junction section matching. This is resolved using a set of rules detailed in the SI.

### Optimization Method

Given its status as a high quality and expensive writing medium, parchment should have an approximately flat surface and uniform thickness for writing. Despite being scuffed and distorted to some extent, most of the parchment layers can still be considered approximately locally uniform. Accordingly, based on this condition, supposing there are *n* curves in **C**_*i*_, our segmentation method will circularly optimize these curves to separate the fused region of the parchment into several layers as evenly as possible.

In order to optimize the curve *r* in **C**_*i*_, 1 ≤ *r* ≤ *n*, we first separate the foreground of ${{\bf{\text{S}}}}^{0}{}_{i}$ into several layers by all the curves except the curve *r*, and then calculate the distance transform **D** of this intermediate segmentation result in such a way that each pixel in **D** is assigned the distance between that pixel and the nearest background pixel. Then the shape energy map **E** of ${{\bf{\text{S}}}}^{0}{}_{i}$ can be calculated by subtracting each pixel value of **D** from the maximum value of **D**

2 $${{\bf{\text{E}}}}_{pq}={d}_{max}-{{\bf{\text{D}}}}_{pq},$$

in which **D**_*pq*_ (**E**_*pq*_) denotes the pixel on the *p*th row and *q*th column in **D**(**E**), and *d*_*max*_ means the maximum value of **D**. It is clear that the shape energy becomes relatively higher at the place closer to the layer boundary and reaches the minimum at the central line of the region.

Let **p**(*k*) = [*x*(*k*) *y*(*k*)]^*T*^ be the parametric equation of the curve *r*, *k* ∈ [0, *m*], where *m* is the length of the curve *r*. On condition that the two endpoints **p**(0) = [*x*_*o*_*y*_*o*_]^*T*^ and **p**(*m*) = [*x*_*e*_*y*_*e*_]^*T*^ are fixed, then the curve *r* coincides with the central line of the region as much as possible only if this curve minimizes the following equation

3 $$\begin{array}{c}minf(x,y)={\int }_{0}^{m}{\bf{E}}(x(k),y(k))dk\\ {\textstyle \text{s.t.}}\\ x(0)={x}_{o},y(0)={y}_{o},x(m)={x}_{e},y(m)={y}_{e}.\end{array}$$

However, if the region is irregular, the curve obtained from Eq. (3) will not be smooth enough, particularly at the endpoints **p**(0) and **p**(*m*). Therefore, we will smooth the curve *r* by constraining its curvature at each point. Since the 2-norm of the second derivative of the curve at a point will be equal to its curvature at this point if the curve is parameterized by arc length^22^, the final cost function can be formulated as follows.

4 $$\begin{array}{c}minf(x,y)={\int }_{0}^{m}\parallel {\bf{p}}(k{)}^{^{\prime\prime} }{\parallel }^{2}dk+\beta {\int }_{0}^{m}{\bf{E}}(x(k),y(k))dk\\ {\textstyle \text{s.t.}}\\ x(0)={x}_{o},y(0)={y}_{o},x(m)={x}_{e},y(m)={y}_{e},\end{array}$$

in which *β* is the weight making the tradeoff between the smoothness and the external force. Further details of the algorithm for performing the optimization are provided in the SI.

The updated curve *r* is substituted for the old curve *r* in **C**_*i*_. All the curves will be optimized circularly until the segmentation converges. Generally, the process of the convergence of segmentation can be very time-consuming, but in practice, we find that the segmentation result changed very slightly after three cycles, so in our work, we just optimize all the curves in **C**_*i*_ for three cycles.

### Layer Connection and Final Segmentation

**Figure 9:**
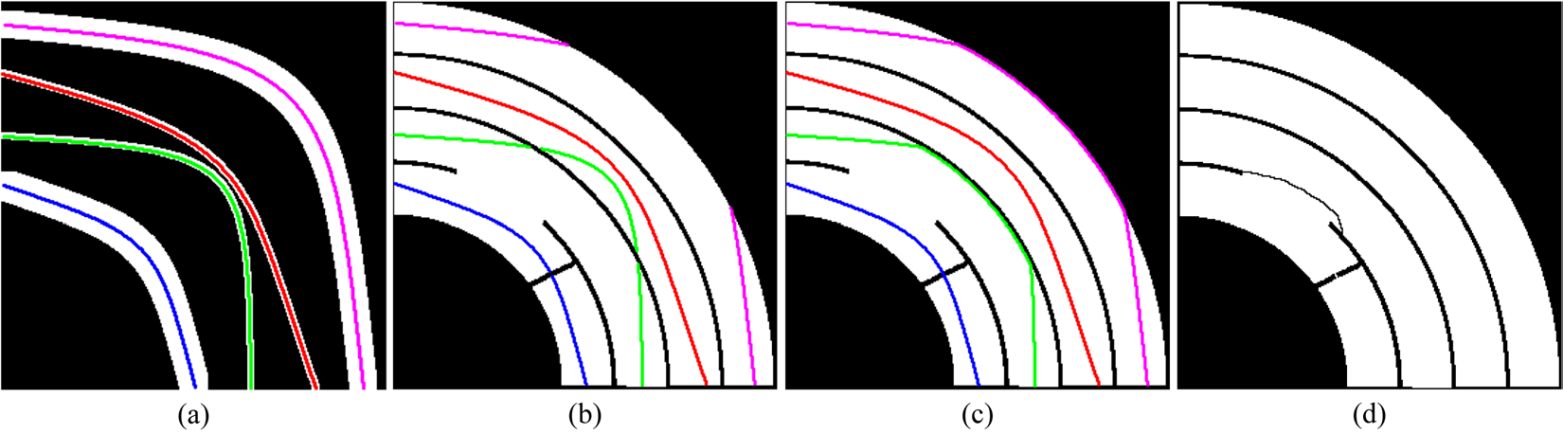
The layer connection for a parchment containing multiple sheets and the final segmentation result. (**a**) The final segmentation **B**_*i*−1_ of the slice *i*−1 and its skeletons; (**b**) the currently-obtained segmentation **B**_*i*_ of the slice *i* and the skeletons of **B**_*i*−1_ contained in the foreground of **B**_*i*_; (**c**) the final skeleton linking result, and (**d**) the final segmentation result.

In this section, we need to refine the already-obtained segmentation and link the broken layer parts together to generate a final segmentation for the ink projection. Given the great similarity between two adjacent images, we can adopt the previous skeleton to determine which two layer parts should be linked in the current image. Figure 9 demonstrates performing layer connection for a parchment consisting of *s* = 4 multiple sheets. In Fig. 9a is shown the final segmentation **B**_*i*−1_ of the slice *i*−1 as well as its skeleton image **K**_*i*−1_, in which the skeletons of different sheets are represented by different colors. The white pixels in Fig. 9b are the foreground of the currently obtained segmentation **B**_*i*_ of the slice *i*, where the shortest sheet is broken and merged with its adjacent sheet. The skeletons of **B**_*i*−1_ which are contained in the foreground of **B**_*i*_ are computed by

5 $${{\bf{U}}}_{i}={{\bf{K}}}_{i-1}\cap {{\bf{B}}}_{i},$$

and superimposed upon the foreground of **B**_*i*_ in Fig. 9b. Obviously, the layer shape of **B**_*i*−1_ is dramatically different from the layer shape of **B**_*i*_, so it poses a big challenge to our method for extracting each sheet from **B**_*i*_ using **B**_*i*−1_. We can link the broken skeletons in **U**_*i*_ using the layer boundary of **B**_*i*_, the convex hull of **B**_*i*_ as well as the background skeleton of **B**_*i*−1_ (see details in SI). The connection result is illustrated in Fig. 9c.

After getting the complete skeletons, we treat each skeleton as a set of seeds and grow them to generate the final segmentation result. Figure 9d demonstrates the final segmentation result. Accordingly, each single sheet can be obtained from Fig. 9d. The technical details of this growth process are given in the SI.

### Ink Projection

Each extracted skeleton is mapped to a row in the output image, and adjacent skeletons are aligned using dynamic programming to minimise spatial distortion. Since the ink used for writing on the parchments was dense it appears as large values in the XMT images. To reconstruct the ink we perform a maximum projection of the intensities along the normals to the skeletons^19^, either in both directions for single-sided documents, or separately in each direction for double-sided documents, creating two output images. The flattening algorithm does not assume the parchment surface to be developable. Instead, it tries to align skeletons to maximize geometric closeness of aligned skeleton points between adjacent slices while minimizing stretching of each skeleton during flattening.

### Code availability

The source code that was used to perform the virtual unrolling is available upon request.

### Data availability

All data are stored on Queen Mary University of London’s server and are available upon request.

## Electronic supplementary material

Supplementary Information

## Footnotes

1 For convenience, we refer to ‘curve segments’ as ‘curves’ without ambiguity.
