# Supplementary material for: Virtual Recovery of Content from X-Ray Micro-Tomography Scans of Damaged Historic Scrolls
Source: Sci Rep. 2018 Aug 9;8:11901. doi: 10.1038/s41598-018-29037-x (PMC6085331; doi:10.1038/s41598-018-29037-x)
Supplement: Supplementary file 1 — Supplementary Information [file 41598_2018_29037_MOESM1_ESM.pdf]

Supplementary Material

Virtual Recovery of Content from X-Ray Micro-Tomography Scans of Damaged Historic Scrolls

Paul L. Rosin[1*], Yu-Kun Lai[1], Chang Liu[2], Graham R. Davis[3], David Mills[3], Gary Tuson[4], and Yuki Russell[4]

[1]School of Computer Science & Informatics, Cardiff University, Queens Buildings, 5 The Parade, Cardiff CF24 3AA, UK
[2]School of Astronautics, Beihang University, Beijing 100191, China
[3]Institute of Dentistry, Queen Mary University of London, Francis Bancroft Building, Mile End Road, London E1 4NS, UK
[4]Norfolk Record Office, The Archive Centre, Martineau Lane, Norwich, NR1 2DQ, UK
[*]Paul.Rosin@cs.cf.ac.uk

Skeleton Propagation between Images

Since adjacent images are very similar, we propagate the segmentation of one slice as an initialisation of the segmentation of the adjacent slice.

Given the final segmentation $\mathbf{S}_{i-1}$ of the slice $i-1$ and the initial segmentation $\mathbf{S}^0{}_i$ of the slice i generated by Otsu method, the skeleton of the background of $\mathbf{S}_{i-1}$ which is included in the foreground of $\mathbf{S}^0{}_i$ can be calculated by Boolean intersection

$$\mathbf{C}_i = skeleton(\bar{\mathbf{S}}_{i-1}) \cap \mathbf{S}^0{}_i, \tag{1}$$

where $skeleton(.)$ represents the morphological skeleton extraction, and $\bar{\mathbf{S}}_{i-1}$ denotes the background of $\mathbf{S}_{i-1}$. The obtained curves in $\mathbf{C}_i$ are superimposed upon $\mathbf{S}^0{}_i$. A pair of junction sections which should be matched are close to a curve in $\mathbf{C}_i$. Therefore, the curves in $\mathbf{C}_i$ can be used to match the pairs of junction sections which should be connected, and will provide an initial guess for the final segmentation.

However, the narrow fused region in $\mathbf{S}^0{}_i$ may obstruct the junction section matching. Fig. 1(a) presents the segmentation $\mathbf{S}_{i-1}$ of slice $i-1$ as well as its foreground skeleton (red curve) and background skeleton (black curve), and Fig. 1(b) shows the initial segmentation $\mathbf{S}^0{}_i$ of slice i. The background skeleton of $\mathbf{S}_{i-1}$ contained in the foreground of $\mathbf{S}^0{}_i$ is displayed in Fig. 1(c). In this subfigure, the two junction sections between the two fused layers cannot be matched, because the background skeleton of $\mathbf{S}_{i-1}$ between these two junction sections cannot be entirely contained in the narrow fused region, and is thereby broken by Eq.(1). To tackle this problem, we exploit the foreground skeleton of $\mathbf{S}_{i-1}$ to identify which two curves in $\mathbf{C}_i$ should be connected together. In Fig. 1(d), the red curve $\mathbf{c}$ with $\mathbf{p}_1$ and $\mathbf{p}_2$ as endpoints is a foreground skeleton of $\mathbf{S}_{i-1}$ included in the background of $\mathbf{S}^0{}_i$. We link $\mathbf{p}_1$ and $\mathbf{p}_2$ by the shortest path $\mathbf{t}$ (red dotted line in Fig. 1(e)) along the layer boundary. If $\mathbf{t}$ only touches two endpoints of two different curves in $\mathbf{C}_i$ and also satisfies $\frac{1}{\alpha} \leq \frac{length\ of\ \mathbf{t}}{length\ of\ \mathbf{c}} \leq \alpha$, these two curves will be linked by the shortest path along the layer boundary, as illustrated in Fig. 1(f). In our work, α is set as 5.

In reality, not all the curves in $\mathbf{C}_i$ are useful for the segmentation process, so we will remove those extraneous curves from $\mathbf{C}_i$. For each endpoint of a curve in $\mathbf{C}_i$, we try to find in $\mathbf{S}^0{}_i$ the junction section which meets the following three conditions: (i) the junction section is within l_1 pixels away from the curve; (ii) the distance between the junction section and the endpoint is less than l_2 pixels, and (iii) the junction section is nearest to the endpoint among all the junction sections meeting conditions (i) and (ii). In our work, l_1 and l_2 are set as 3 and 20 respectively. The pair of junction sections corresponding to the same curve is considered to be matched by this curve. From $\mathbf{C}_i$ will be deleted the curves which cannot match two junction sections and are shorter than a threshold d (100 in our work), because these curves are too short and useless.

The obtained useful curves in $\mathbf{C}_i$ will be finally preprocessed as the initial guess for the further optimization. If the endpoint $\mathbf{e}$ of the curve has a corresponding junction section, whose middle point $\mathbf{m}$ is closest to the point $\mathbf{e}'$ of the curve (Fig. 2(a)), then we will cut out the segment $\mathbf{ee}'$ and link $\mathbf{m}$ and $\mathbf{e}'$ together by a straight line segment. On the other hand, if $\mathbf{e}$ has no

corresponding junction section, as shown in Fig. 2(b), then let the boundary point $\mathbf{q}$ be the nearest to $\mathbf{e}$, and we will link $\mathbf{q}$ and $\mathbf{e}$ by a straight line segment.

Optimization Method for Even Segmentation

The main body of the paper formulated the goal of optimizing a set of boundary curves in order to separate a fused region into several layers as evenly as possible. Details are provided in the SI of how Eq. (4) is solved, and the method is then demonstrated on a simple synthetic example.

Optimization Method

The necessary condition of Eq. (4) requires $\mathbf{p}$ to satisfy the Euler-Lagrange equation[1]

$$2\frac{d^4}{dk^4}\mathbf{p}(k)+\beta\nabla_{\mathbf{p}}\mathbf{E}(\mathbf{p}(k))=0. \tag{2}$$

The traditional method to solve Eq. (2) first treats $\mathbf{p}$ as a function of k as well as time t, and then sets the negative partial derivative of $\mathbf{p}$ with respect to t equal to the left side of Eq. (2)

$$-\frac{d}{dt}\mathbf{p}(k,t)=2\frac{d^4}{dk^4}\mathbf{p}(k,t)+\beta\nabla_{\mathbf{p}}\mathbf{E}(\mathbf{p}(k,t)), \tag{3}$$

and finally solves Eq. (3) by finite difference (FD)[2]. In the practical application of FD, if $\triangle k$ is fixed, in order to stabilize the solution $\mathbf{p}(k,t)$, the temporal step $\triangle t$ has to be carefully chosen to make the numerical scheme satisfy the Courant-Friedrichs-Lewy condition[3]. However, it is difficult to find a $\triangle t$ suitable for all different kinds of parchment scrolls, due to the fact that these scrolls are quite different in the shape of layer, image size, and gray level and so on. Therefore, for the adaptability of the algorithm, we will treat Eq. (4) as nonlinear optimization. Discretizing Eq. (4) yields

$$\min f(\mathbf{p}_2,\ldots,\mathbf{p}_{h-1})=\sum_{j=1}^{h}\|\mathbf{p}_{j-1}-2\mathbf{p}_j+\mathbf{p}_{j+1}\|^2\frac{1}{\triangle k^3}+\beta\sum_{j=1}^{h}\mathbf{E}(\mathbf{p}_j)\triangle k \tag{4}$$

s.t.

$$x_1=x_o, y_1=y_o, x_h=x_e, y_h=y_e,$$

in which, the point $\mathbf{p}_j$ corresponds to the point $\mathbf{p}((j-1)\triangle k)$, and $\mathbf{p}_h=\mathbf{p}(m)$. Because $\triangle k$ is fixed, Eq. (4) is equivalent to the following minimization

$$\min f(\mathbf{p}_2,\ldots,\mathbf{p}_{h-1})=\sum_{j=1}^{h}\|\mathbf{p}_{j-1}-2\mathbf{p}_j+\mathbf{p}_{j+1}\|^2+\beta'\sum_{j=1}^{h}\mathbf{E}(\mathbf{p}_j) \tag{5}$$

s.t.

$$x_1=x_o, y_1=y_o, x_h=x_e, y_h=y_e,$$

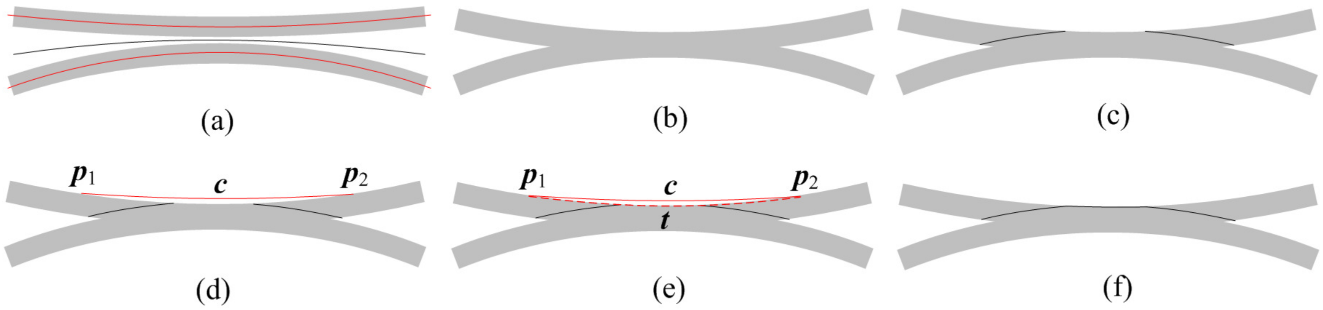

Figure 1. The connection of the curves in $\mathbf{C}_i$. (a) The foreground skeleton (red curve) and the background skeleton (black curve) of $\mathbf{S}_{i-1}$; (b) the initial segmentation $\mathbf{S}^0{}_i$; (c) the background skeleton segment of $\mathbf{S}_{i-1}$ is broken between the two junction sections of the fused region in $\mathbf{S}^0{}_i$; (d), (e), and (f) determine which two curves in $\mathbf{C}_i$ should be linked by means of the foreground skeleton of $\mathbf{S}_{i-1}$ included in the background of $\mathbf{S}^0{}_i$.

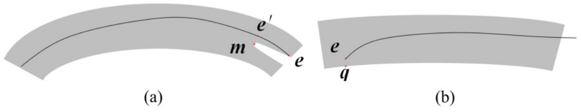

Figure 2. The preprocessing of curves in $\mathbf{C}_i$.

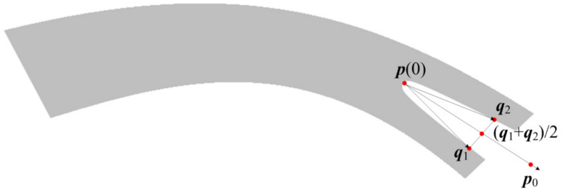

Figure 3. The construction of $\mathbf{p}_0$.

where $\beta' = \beta\triangle k^4$. The two additional points $\mathbf{p}_0$ and $\mathbf{p}_{h+1}$ in Eq. (5) can be constructed according to the shape of the junction sections where $\mathbf{p}(0)$ and $\mathbf{p}(m)$ are respectively located. We take $\mathbf{p}_0$ as an example to explain the construction method. As demonstrated in Fig. 3, given that $\mathbf{p}(0)$ is an endpoint of curve r, and $\mathbf{q}_1$ and $\mathbf{q}_2$ are two boundary points which are w pixels away from and at both sides of $\mathbf{p}(0)$, $\mathbf{p}_0$ can be determined by

$$\mathbf{p}_0 = \frac{l(0.5(\mathbf{q}_1+\mathbf{q}_2)-\mathbf{p}(0))}{\|0.5(\mathbf{q}_1+\mathbf{q}_2)-\mathbf{p}(0)\|}, \tag{6}$$

where l is the distance between $\mathbf{p}_0$ and $\mathbf{p}(0)$. Larger values of l will make the curve r smoother at the endpoint $\mathbf{p}(0)$. We set l as 10 and w as 7 in our work. $\mathbf{p}_{h+1}$ can be decided in the same way.

By convention, Eq. (5) can be further rewritten into matrix form

$$\min f(\mathbf{X}) = \mathbf{X}^T\mathbf{M}\mathbf{X} + \mathbf{P}^T\mathbf{X} + Q + \beta' g(\mathbf{X}), \tag{7}$$

where $\mathbf{X} = [x_2 \cdots x_{h-1}\ y_2 \cdots y_{h-1}]^T$, $M = \begin{bmatrix} \mathbf{A} & \mathbf{0} \\ \mathbf{0} & \mathbf{A} \end{bmatrix}$, in which $\mathbf{A}$ is a pentadiagonal matrix in the form

$$\mathbf{A} = \begin{bmatrix} 6 & -4 & 1 & & & & \\ -4 & 6 & -4 & \ddots & & \mathbf{0} & \\ 1 & -4 & 6 & & & & \\ & \ddots & & \ddots & & \ddots & \\ & & & & 6 & -4 & 1 \\ & \mathbf{0} & & \ddots & -4 & 6 & -4 \\ & & & & 1 & -4 & 6 \end{bmatrix}$$, and $\mathbf{P} = \begin{bmatrix} \mathbf{G}_1 & \mathbf{G}_2 \end{bmatrix}^T$ with $\mathbf{G}_1$ and $\mathbf{G}_2$ as

$\mathbf{G}_1 = \begin{bmatrix} 2x_0 - 8x_1 & 2x_1 & 0 & \cdots & 0 & 2x_h & 2x_{h+1} - 8x_h \end{bmatrix}$; $\mathbf{G}_2 = \begin{bmatrix} 2y_0 - 8y_1 & 2y_1 & 0 & \cdots & 0 & 2y_h & 2y_{h+1} - 8y_h \end{bmatrix}$; Q is a constant expressed as $Q = {x_0}^2 + 5{x_1}^2 - 4x_0x_1 + {x_{h+1}}^2 + 5{x_h}^2 - 4x_hx_{h+1} + {y_0}^2 + 5{y_1}^2 - 4y_0y_1 + {y_{h+1}}^2 + 5{y_h}^2 - 4y_hy_{h+1} + \beta'\mathbf{E}(x_1,y_1) + \beta'\mathbf{E}(x_h,y_h)$, and finally $g(\mathbf{X}) = \sum_{j=2}^{h-1}\mathbf{E}(x_j,y_j)$. We use the steepest descent method to optimize Eq. (7). The gradient of $f(\mathbf{X})$ can be produced by

$$\nabla_{\mathbf{X}} f = 2\mathbf{M}\mathbf{X} + \mathbf{P} + \beta'\nabla_{\mathbf{X}} g(\mathbf{X}). \tag{8}$$

Thus $\mathbf{X}^{(t+1)}$ in tth iteration can be updated by

$$\mathbf{X}^{(t+1)} = \mathbf{X}^{(t)} - \lambda\nabla_{\mathbf{X}} f = (\mathbf{I} - 2\lambda\mathbf{M})\mathbf{X}^{(t)} - \lambda\mathbf{P} - \lambda\beta'\nabla_{\mathbf{X}} g^{(t)}, \tag{9}$$

where $\mathbf{I}$ denotes identity matrix, and λ is the step-size along the direction of the gradient, automatically chosen by Armijo backtracking, and $\nabla_{\mathbf{X}} g^{(t)} = \begin{bmatrix} \nabla_{x_2}\mathbf{E}({x_2}^{(t)},{y_2}^{(t)}) & \cdots & \nabla_{y_{h-1}}\mathbf{E}({x_{h-1}}^{(t)},{y_{h-1}}^{(t)}) \end{bmatrix}^T$. With the fourth order central difference, $\nabla_{x_j}\mathbf{E}(x_j,y_j)$ and $\nabla_{y_j}\mathbf{E}(x_j,y_j)$ can be respectively calculated by

$$\nabla_{x_k}\mathbf{E} = \frac{-\mathbf{E}(x_k+2,y_k) + 8\mathbf{E}(x_k+1,y_k)}{12} - \frac{8\mathbf{E}(x_k-1,y_k) + \mathbf{E}(x_k-2,y_k)}{12}, \tag{10}$$

$$\nabla_{y_k}\mathbf{E} = \frac{-\mathbf{E}(x_k, y_k+2) + 8\mathbf{E}(x_k, y_k+1)}{12} - \frac{8\mathbf{E}(x_k, y_k-1) + \mathbf{E}(x_k, y_k-2)}{12}. \tag{11}$$

It can be easily proved that the update formula (9) has the same form as the explicit finite difference discretization of Eq.(3) has. However because of dynamically choosing the step-size λ, the proposed method does not suffer from the aforementioned drawback of the FD.

As mentioned in the main body of this paper, all n curves in $\mathbf{C}_i$ will be sequentially optimized for three cycles by this method, since we find that this provides sufficient convergence. The pseudo-code of our optimization method is described in ALGORITHM 1 as follows.

ALGORITHM 1: Optimization

Input: n curves propagated from previous segmentation results.
For *cycle*=1 to 3
  For r=1 to n
    Get segmentation result by all the curves except curve r.
    Generate the shape energy map $\mathbf{E}$ by Eq. (2).
    Calculate $\mathbf{p}_0$ and $\mathbf{p}_{h+1}$ by Eq. (6).
    Optimize the curve r by Eq (7).
    Replace the old curve r by the updated one.
  End
End
Output: n optimized curves.

Experiment on Synthetic Data

An experiment is performed to verify the effectiveness of the proposed optimization algorithm. In this experiment, we set β' as 300. Fig. 4(a) illustrates a fused region which is comprised of 4 layers. Fig. 4(b) demonstrates the initial guess of the three curves which link the matched junction sections to separate the fused region. Obviously, the quality of the initial guess is fairly bad since not only are these curves quite irregular but they do not evenly separate the fused region at all. In order to update one curve, we first use the other curves to segment the fused region, getting segmentation $\mathbf{T}$, as shown in Fig. 4(c). The shape energy map $\mathbf{E}$ of $\mathbf{T}$ is shown in Fig. 4(d), in which the brighter pixels correspond to higher energy. It can be noticed that the local minima in $\mathbf{E}$ are located in the middle of each region. The optimization result of that curve is displayed in Fig. 4(e). It can be seen that the optimized curve (blue) almost lies on the middle of the region and is much smoother than its initial guess (red). The final segmentation result is presented in Fig. 4(f). As compared with Fig. 4(b), it evident that in spite of the bad initial guess of these curves, the optimization algorithm still provides a high-quality segmentation result, which demonstrates the effectiveness and robustness of our method.

Broken Skeleton Connection and Final Segmentation

We now detail how to connect the broken skeletons in $\mathbf{U}_i$ and the growth process used to get the final segmentation. Let $\mathbf{f}_i$ be the layer boundary of $\mathbf{B}_i$, then the endpoints of the skeleton segments in $\mathbf{U}_i$ must lie on the boundary $\mathbf{f}_i$, as shown in Fig. 5(a). Therefore, we can first link the broken skeletons in $\mathbf{U}_i$ along the boundary $\mathbf{f}_i$. Supposing that a sheet skeleton of $\mathbf{B}_{i-1}$ has been broken into m parts in $\mathbf{U}_i$, which gives rise to $2m$ endpoints, we number these endpoints in order along the skeleton, as exhibited in Fig. 5(a). It can be seen that a point with an even number may only be connected to a point with an odd number larger than that even number, for example, the point 2 can only be linked with point 3 or point 5. Accordingly, given an even number point r and an odd number point s, where $r = 2, 4, \dots, 2(m-1)$ and $s = r+1, r+3, \dots, 2m-1$, we respectively find in $\mathbf{f}_i$ and $\mathbf{K}_{i-1}$ the shortest paths $\mathbf{p}^f{}_{rs}$ and $\mathbf{p}^K{}_{rs}$ between these two points by Dijkstra's algorithm. If $\mathbf{p}^f{}_{rs}$ does not intersect with any of the skeleton part in $\mathbf{U}_i$, and $\mathbf{p}^f{}_{rs}$ and $\mathbf{p}^K{}_{rs}$ meet the relationship $\frac{1}{\rho} \leq \frac{length\ of\ \mathbf{p}^f{}_{rs}}{length\ of\ \mathbf{p}^K{}_{rs}} \leq \rho$, we will delete the pixels of $\mathbf{p}^K{}_{rs}$ in $\mathbf{U}_i$, and then link the endpoints r and s by $\mathbf{p}^f{}_{rs}$ in $\mathbf{U}_i$; we subsequently proceed to check the even number point $s+1$. Otherwise, the point $r+2$ will be checked by the same way. ρ is set to 3 in our work. The connection result is illustrated in Fig. 5(b).

Note that the skeleton segments contained in the three longer sheets have been correctly connected. In order to finally link the skeleton segments in the shortest broken sheet, we first calculate the convex hull $\mathbf{H}_i$ of $\mathbf{B}_i$, and then obtain the Boolean union $\mathbf{L}_i$ of $\mathbf{H}_i$, the background skeleton of $\mathbf{B}_{i-1}$, and $\mathbf{U}_i$. $\mathbf{L}_i$ is provided in Fig. 5(c), where the convex hull $\mathbf{H}_i$ and the background skeleton of $\mathbf{B}_{i-1}$ are respectively represented by the brown and black curves. Fig. 5(c) indicates that $\mathbf{L}_i$ gives the borders to the gap between the broken skeleton parts. On the constraint of such borders, the curves which link the broken parts will be regular

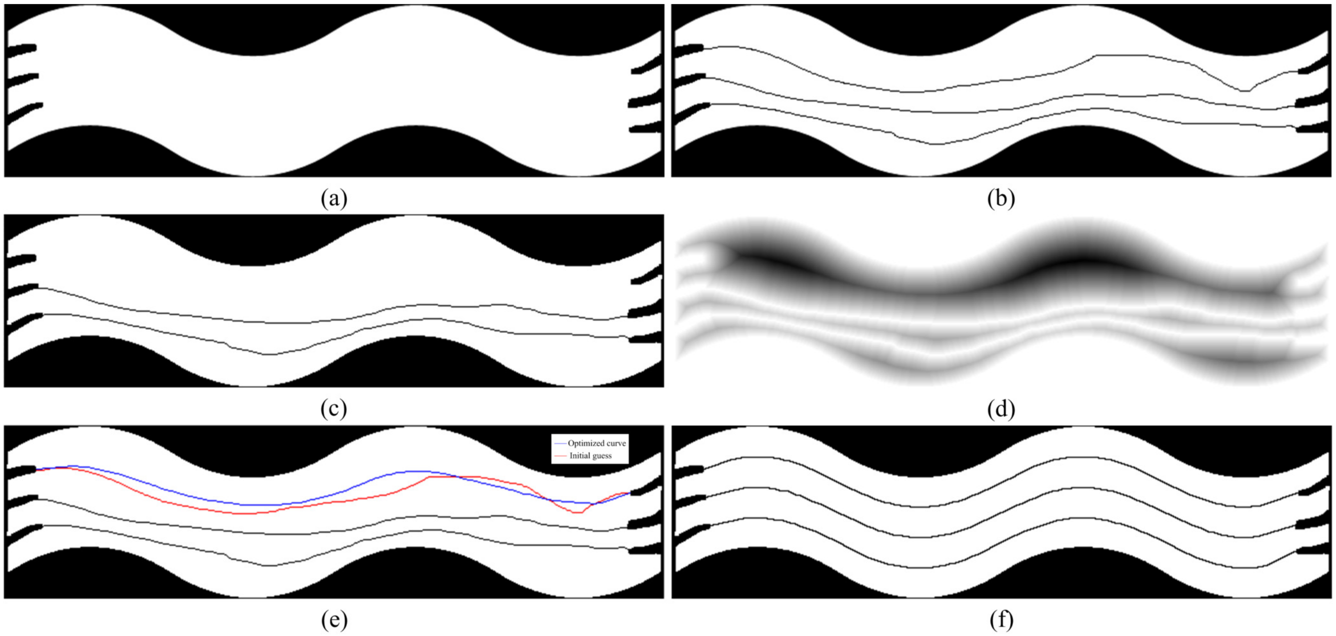

Figure 4. Curve optimization. (a) A fused region comprised of 4 layers; (b) the segmentation by the initial guess of the curves; (c) the segmentation by all curves except the one to be optimized; (d) the shape energy map of Fig. 4(c); (e) the optimization result of the first curve, and (f) the final optimization result.

and prevented from intersecting with other skeletons. For a sheet skeleton broken into v parts in $\mathbf{U}_i$, we number these endpoints in order along the skeleton, as illustrated in Fig. 5(c). It can be observed that each even number point r should be linked with point $r+1$, $r = 2, 4, \ldots, 2(v-1)$. Therefore we first obtain the shortest path $\mathbf{p}^K{}_{r(r+1)}$ between points r and $r+1$ from $\mathbf{K}_i$; then fix the endpoints of $\mathbf{p}^K{}_{r(r+1)}$, and meanwhile push $\mathbf{p}^K{}_{r(r+1)}$ away from the borders by the optimization method (see Section C in the main manuscript) with the shape energy map of the non-border region in $\mathbf{L}_{i+1}$. The final linking result is illustrated in Fig. 5(d).

After getting the complete skeletons, we treat each skeleton as a set of seeds and grow them to generate the final segmentation result. Let $\mathbf{F}_i$ be the foreground of $\mathbf{B}_i$ and R_j be the set of seed points initially formed by the skeleton j in $\mathbf{U}_i$, $1 \le j \le s$, if a point $\mathbf{p}$ which is 8-connected to R_j satisfies the condition: $\mathbf{p} \subseteq \mathbf{F}_i$, $\mathbf{p} \nsubseteq R_j$ and $\mathbf{p}$ is not adjacent to any other sets of seeds except R_j, then this point will be added to R_j. We add all of R_j's adjacent points that satisfy the condition to R_j, and take the grown set R_j as a new set of seeds. We repeatedly grow all the sets of seeds one by one until no point should be added to the seed sets. Fig. 6 demonstrates some intermediate results during growing (Fig. 6(a)-(c)) and the final segmentation result (Fig. 6(d)). Accordingly, each single sheet can be obtained from Fig. 6(d).

Experiment on Real Scroll Data

Several experiments are performed to test the accuracy of the approach. The first experiment is conducted to deal with the Bressingham scroll. β' in Eq. (5) is set as 200 here. Fig. 7(a) illustrates a typical XMT image of the parchment, whose initial segmentation is shown in Fig. 7(b). It can been seen from Fig. 7(a) and (b) that there are a large number of missing parts of the layers, and furthermore, the fused region consisting of 4 layers is compressed, and is only around 10 pixels wide. We use our algorithm to separate the fused regions into several layers and connect the broken layers together. The result is shown in Fig. 7(c), where the red box highlights the segmentation of a fused region consisting of 4 layers. Although the narrowest part of this region is only about 10 pixels wide, our method still evenly separates it into four layers, which strongly testifies to the effectiveness of our method. In addition, it should also be noticed that curves linking the broken layers are equally spaced in the background of the image, thus avoiding intersecting with each other. The segmentation obtained by Liu *e*t al.'s method[4] is illustrated in Fig. 7(d). As compared to our result (Fig. 7(c)), there exist some long and thin fused regions which have not been segmented.

The next experiment is to segment the X-ray image of the Diss Heywood burnt scroll which consists of four sheets. β' in Eq. (5) is set as 300 in this trial. All the sheets are fused together in this scroll, and it is therefore a great challenge for our method to segment the four different sheets out of the XMT image. Fig. 8(a) presents a tomographic slice of the scroll. The big

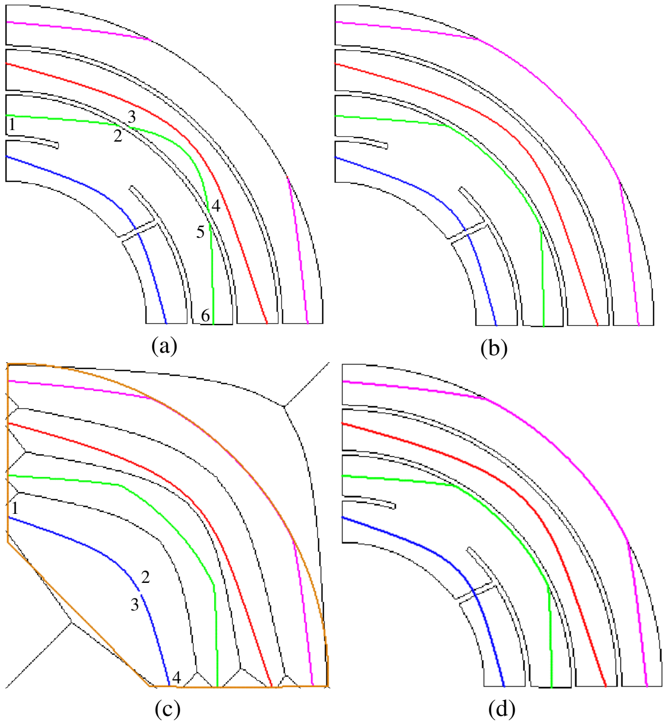

Figure 5. The layer connection for a parchment containing multiple sheets. (a) The layer boundary $\mathbf{f}_i$ of $\mathbf{B}_i$, on which the endpoints are number in order along the skeleton; (b) some broken skeleton parts are connected along the boundary; (c) the Boolean union of the convex hull of $\mathbf{B}_i$, the background skeleton of $\mathbf{B}_{i-1}$, and $\mathbf{U}_i$; (d) the final linking result.

chunks of fused regions can be clearly observed in the initial segmentation (Fig. 8(b)). In spite of this big difficulty, our method can still generate the correct segmentation for the scroll, as shown in Fig. 8(c). The red and green boxes at the top left corner and the bottom right corner show the corresponding portions of the segmentation result. It can be seen that the fused region has been evenly separated into as many as fifteen regular layers. Fig. 8(d) gives the segmentation result of Liu *e*t al.'s method. As observed, some long fused regions are not successfully separated into several layers. Fig. 9 displays the four sheets segmented from the Fig. 8(b), which clearly demonstrates the correctness and effectiveness of our segmentation method.

Correcting Striping Artifacts

Since the scrolls were scanned as a series of separate volumes it was necessary to perform intensity correction, as otherwise striping artifacts are clearly visible from the multiple volumes being merged together. This was carried out by normalising each row, where a row corresponds to the reconstruction from a single image. Since the majority of the surface of a scroll does not contain writing, and consequently makes up the parchment surface in the reconstruction, the median of the intensities along a row provides an estimate of the typical parchment intensity value. The median absolute deviation (mad) of the row intensities provides an indication of the contrast. It is calculated as the median of the differences of the intensity values from that median:

$$\text{mad} = \operatorname*{med}_{i=1}^{n} |a_i - \operatorname*{med}_{j=1}^{n} a_j|,$$

where a_i denotes the set of intensities in a row. In practice, for the Bressingham scroll, to improve robustness, only 25% of the pixels were used to estimate the median and mad, namely those towards the top of the scroll, since that portion of the scroll was less damaged and contained less artifacts. The median and median absolute deviation respectively provided the offset and scaling for the intensity normalisation. For an intensity I represented in the range [0,255] the normalised intensity is

$$I' = (I - \text{med}) \times \frac{25}{\text{mad}} + 200.$$

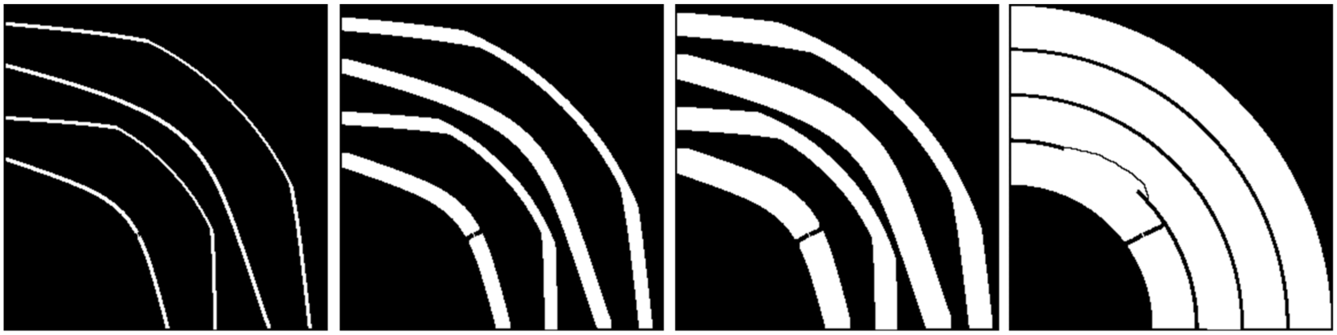

(a) (b) (c) (d)

Figure 6. Skeletons are grown to generate the final segmentation. (a)-(c) The intermediate results. (d) The final result.

References

1. Lao, D. *Fundamentals of the Calculus of Variations*, 87–88 (National Defence Industry Press, 2007).

2. Becker, T. & Kaus, B. *Numerical Geodynamics*, 78–80 (University of Southern California, 2010).

3. Trefethen, L. N. *Finite Difference and Spectral Methods for Ordinary and Partial Differential Equations*, 160–163 (Cornell University, 1996).

4. Liu, C., Rosin, P. L., Lai, Y. K. & Hu, W. Robust virtual unrolling of historical parchment XMT images. *IEEE Transactions on Image Processing* **27**, 1914–1926 (2018).

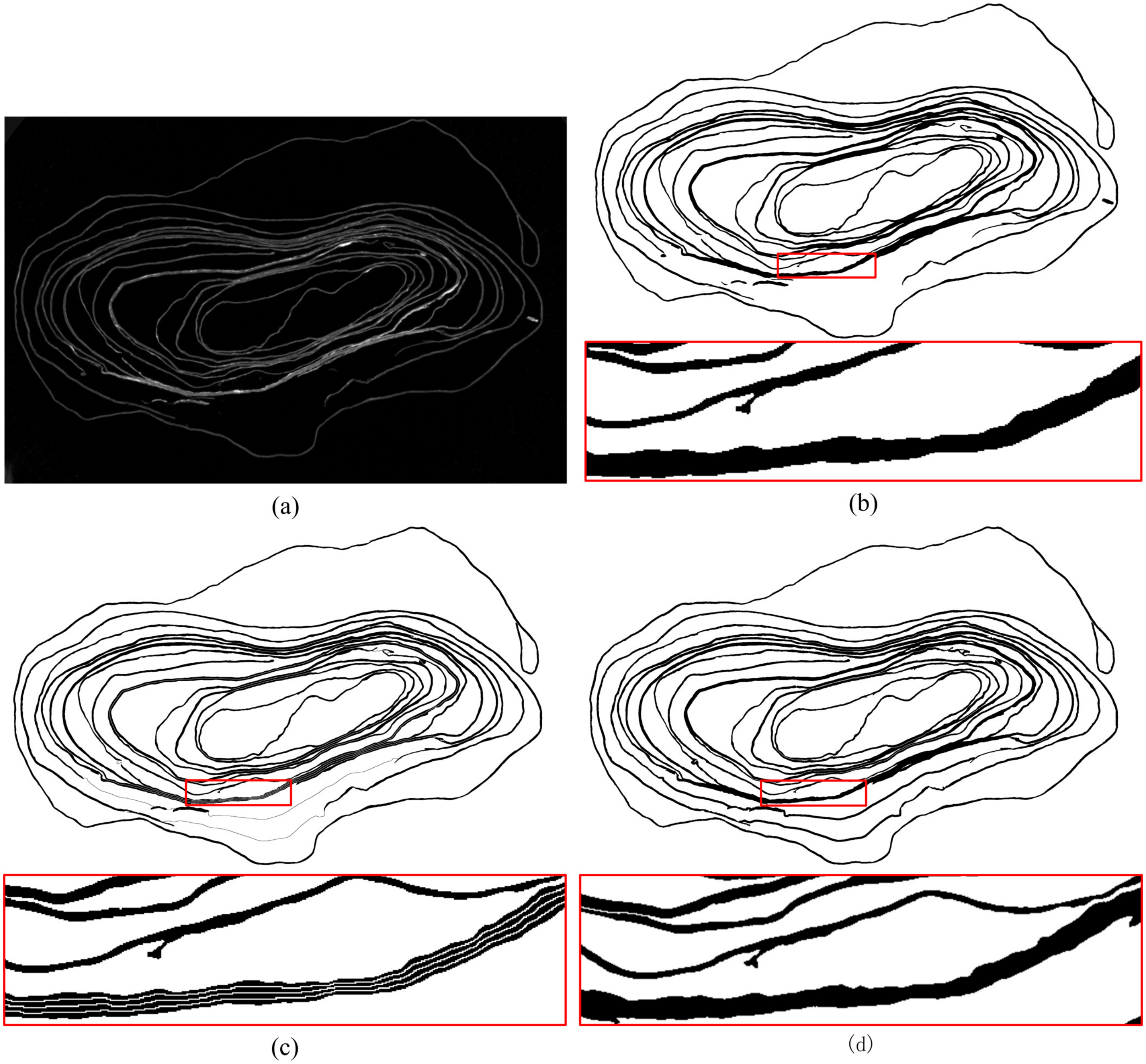

Figure 7. The segmentation of the Bressingham scroll. (a) An XMT image of the Bressingham scroll; (b) the initial segmentation by the Otsu method; (c) the final segmentation of our method; (d) the segmentation result of Liu *et* al.'s method.

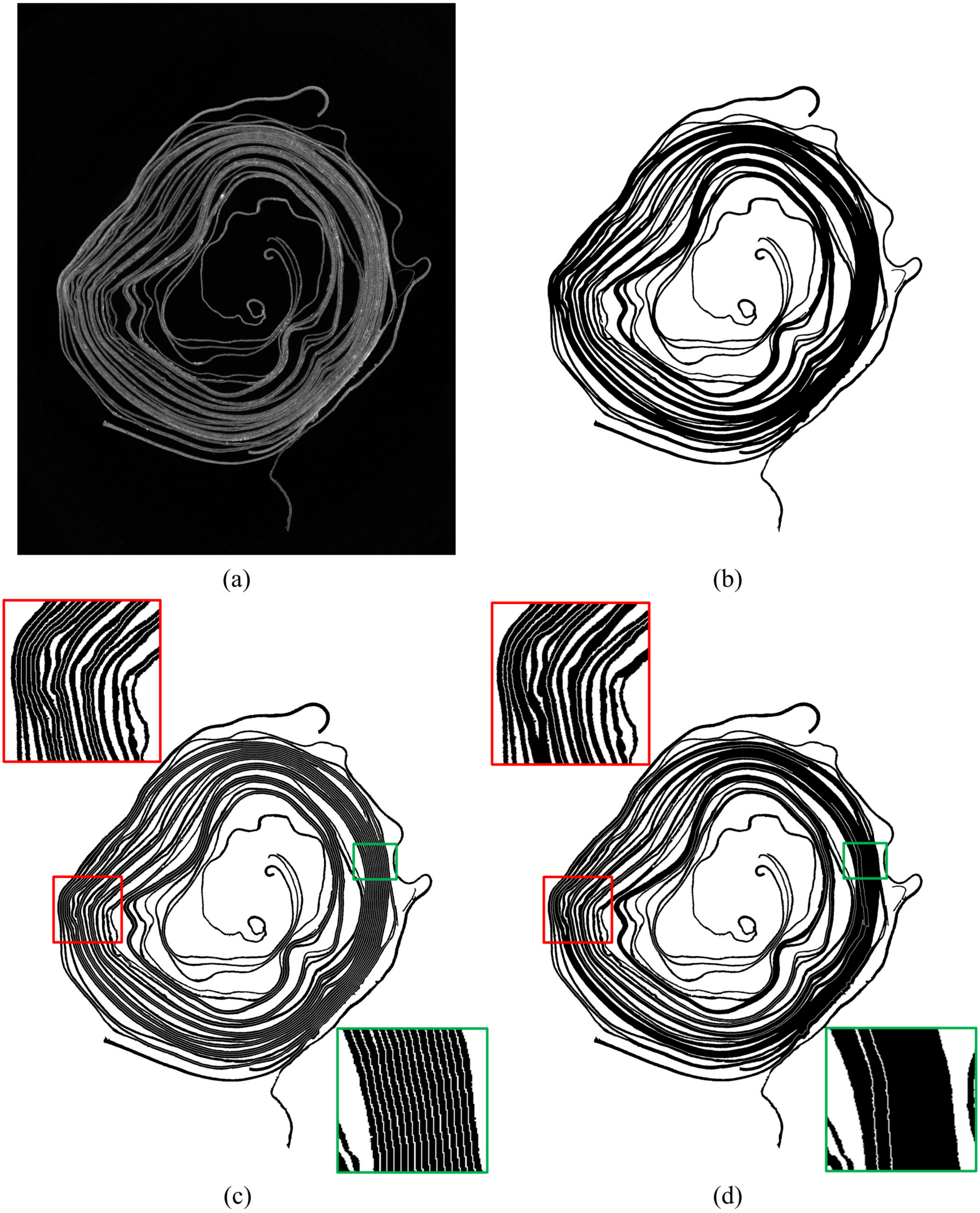

Figure 8. The segmentation of the burnt scroll. (a) An XMT image of the scroll; (b) the initial segmentation by the Otsu method; (c) the final segmentation of our method; (d) the segmentation result of Liu *e*t al.'s method.

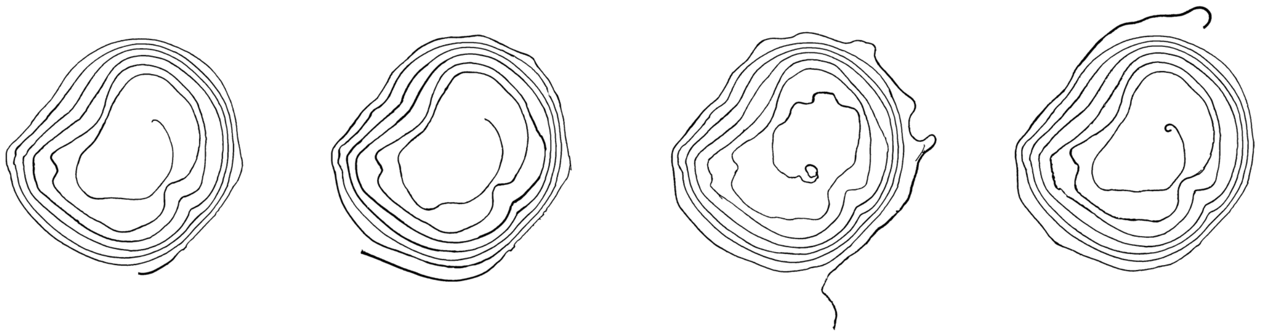

Figure 9. The four sheets segmented out of the original image.
